# Supplementary material for: Association of Breastfeeding and Early Childhood Caries: A Systematic Review and Meta-Analysis
Source: Nutrients. 2024 Apr 30;16(9):1355. doi: 10.3390/nu16091355 (PMC11085424; doi:10.3390/nu16091355)
Supplement: Supplementary file 1 [file nutrients-16-01355-s001.zip › nutrients-2950572-supplementary.pdf]

Supplementary Materials S1

Table S1. Summarised assessment table of previous systematic reviews.

Reference	Search End Date	Research Question/s	Conclusion	Included Study Designs	Databases Searched	Participants Age-Range	AMSTAR Rating
Avila et al. (2015)	March 2015	Association between feeding practice and dental caries in childhood	Breastfeeding is protective against dental caries compared to bottle feeding.	Observational studies (Cohort, case-control and cross-sectional)	PubMed, Cochrane Library, Web of science, Controlled-trials Database of Clinical Trials, Clinical Trials–US National Institute of Health, National Institute for Health and Clinical Excellence, Lilacs	Children ≤ 71 months	Low
Bagher et al. (2013)	March 2011	To conduct a systematic review on association between breastfeeding and dental caries in preschool children in Saudi Arabia	Exclusive breastfeeding, limited nocturnal feeding and early weaning are not associated with ECC	Case-control, cross-sectional	PubMed, Science Direct, Saudi Dental Journal (SDJ), Saudi Medical Journal (SMJ) and Journal of King Abdulaziz University – Medical Sciences.	Preschool children	Low
Cui et al. (2017)	December 2015	To update and summarize the current evidence for association between breastfeeding and ECC	Breastfeeding is protective against ECC. Breastfeeding more than 12 months is not associated with ECC.	Birth cohort, case-control, cross-sectional	PubMed, Embase, Web of science	Children aged 0-71 months	Low
Klaiban et al. (2021)	Not reported	To determine the effect of sufficient breastfeeding and the	Breastfeeding and nocturnal	Comparative studies and experimental	PubMed, Cochrane library, google scholar	Infants	Critically low

		risk of dental caries in infants	breastfeeding were protective against ECC. Consumption of formula increased the risk of ECC.	studies			
Moynihan et al. (2019)	August 2017	What is the best way to maintain health of the primary dentition?	No association between breastfeeding up to 24 months and ECC. Increased risk of ECC if breastfeeding continued beyond 24 months.	RCT, intervention studies, cohort, case-control, ecologic, cross-sectional	Medline, CINAHL, Embase, PubMed, Cochrane Library, PROSPERO	Children <72 months old	Moderate
Sukmana et al. (2020)	20th April 2020	Not reported	Inconclusive	Not reported	PubMed, Google Scholar	Children	Critically low
Tham et al. (2015)	2nd October 2014	To summarise the current evidence for the association between breastfeeding and dental caries with specific reference to exposure windows and breastfeeding practices	Breastfeeding up to 12 months not associated with ECC; it might have protective effect. Breastfeeding >12 months has increased risk of ECC.	Experimental and observational studies	PubMed Central, CINAHL, EMBASE	Children and adolescents	Low
Valaitis et al. (2000)	Not reported	What is the current quality of literature regarding the relationship between ECC and breastfeeding and the association between breastfeeding after eruption of primary teeth and ECC? The effect of duration of breastfeeding on incidence of ECC.	Inconclusive	Cohort, cross-sectional, case-control, case series	Medline, CINAHL, Biological abstract, Social Science index	Children 0-≤4 years	Critically low

* ECC: Early childhood caries

Supplementary Materials S2. Search strategies

MEDLINE (Ovid)

1	exp Breast Feeding
2	(breast Feed* or breastfeed* or breast-feed* or breast fed* or breastfed* or breast-fed*).mp.
3	Milk, Human/
4	(Human milk or breastmilk or breast milk or breast-milk).mp.
5	Lactation/
6	Lactat*.mp.
7	1 or 2 or 3 or 4 or 5 or 6
8	Dental Caries/
9	((Dental* or tooth* or teeth* or oral) adj2 (caries or cario*)).mp.
10	Dental Caries Susceptibility/
11	Tooth Demineralization/
12	(Tooth demineralisation* or tooth demineralization*).mp.
13	Oral Health/
14	Oral health*.mp.
15	Dental health*.mp.
16	(dental adj5 (cavit* or decay* or lesion* or demineral* or reminerali*)).mp.
17	(tooth adj5 (cavit* or decay* or lesion* or demineral* or reminerali*)).mp.
18	(teeth adj5 (cavit* or decay* or lesion* or demineral* or reminerali*)).mp.
19	Early childhood caries.mp.
20	Nursing Bottle Caries.mp.
21	Rampant caries.mp.
22	Baby bottle tooth decay.mp.
23	8 or 9 or 10 or 11 or 12 or 13 or 14 or 15 or 16 or 17 or 18 or 19 or 20 or 21 or 22
24	7 and 23
25	Child, Preschool/
26	(Preschool* or pre-school*).mp.
27	Early childhood*.mp.
28	Toddler*.mp.
29	Infant/
30	Infant*.mp.
31	25 or 26 or 27 or 28 or 29 or 30
32	24 and 31
33	limit 32 to humans

Note:

1. 'exp' denotes exploding the subject heading
2. * symbolises truncation
3. 'mp.' denotes searching keywords at many places in the title and abstract of articles
4. 'adj' denotes two words next to each other in any order

Embase (Ovid)

1	exp breast feeding/
2	(breast Feed* or breastfeed* or breast-feed* or breast fed* or breastfed* or breast- fed*).mp.

3	exp breast milk/
4	(Human milk or breastmilk or breast milk or breast-milk).mp.
5	lactation/
6	Lactat*.mp.
7	1 or 2 or 3 or 4 or 5 or 6
8	dental caries/
9	((Dental* or tooth* or teeth* or oral) adj2 (caries or cario*)).mp.
10	Dental Caries Susceptibility.mp.
11	Tooth Demineralization.mp.
12	(Tooth demineralisation* or tooth demineralization*).mp.
13	Oral Health*.mp.
14	dental health/
15	Dental health*.mp.
16	(dental adj5 (cavit* or decay* or lesion* or demineralі* or reminerali*)).mp.
17	(tooth adj5 (cavit* or decay* or lesion* or demineralі* or reminerali*)).mp.
18	(teeth adj5 (cavit* or decay* or lesion* or demineralі* or reminerali*)).mp.
19	Early childhood caries.mp.
20	Nursing Bottle Caries.mp.
21	Rampant caries.mp.
22	Baby bottle tooth decay.mp.
23	8 or 9 or 10 or 11 or 12 or 13 or 14 or 15 or 16 or 17 or 18 or 19 or 20 or 21 or 22
24	preschool child/
25	(Preschool* or pre-school*).mp.
26	Early childhood*.mp.
27	toddler/
28	toddler*.mp.
29	infant/
30	infant*.mp.
31	24 or 25 or 26 or 27 or 28 or 29 or 30
32	7 and 23 and 31
33	limit 32 to humans

Note:

1. 'exp' denotes exploding the subject heading
2. * symbolises truncation
3. 'mp.' denotes searching keywords at many places in the title and abstract of articles
4. 'adj' denotes two words next to each other in any order

CINHAL (EBSCO)

S1	(MH "Breast Feeding+")
S2	"breast Feed*" or "breastfeed*" or "breast#feed*" or "breast fed*" or "breastfed*" or "breast#fed*"
S3	(MH "Milk, Human+")
S4	"Human milk" or "breastmilk" or "breast milk" or "breast#milk"
S5	(MH "Lactation") OR (MH "Lactates")
S6	Lactat*
S7	S1 OR S2 OR S3 OR S4 OR S5 OR S6
S8	(MH "Dental Caries")

S9	(Dental* or tooth* or teeth* or oral) N2 (caries or cario*)
S10	"Dental Caries Susceptibility"
S11	(MH "Tooth Demineralization")
S12	"Tooth demineralisation" or "tooth demineralization"
S13	(MH "Oral Health")
S14	"Oral health*"
S15	"dental health*"
S16	TI (dental N5 (cavit* or decay* or lesion* or demineralі* or reminerali*)) AB ((dental N5 (cavit* or decay* or lesion* or demineralі* or reminerali*))
S17	TI (tooth N5 (cavit* or decay* or lesion* or demineralі* or reminerali*)) AB ((tooth N5 (cavit* or decay* or lesion* or demineralі* or reminerali*))
S18	TI (teeth N5 (cavit* or decay* or lesion* or demineralі* or reminerali*)) AB ((teeth N5 (cavit* or decay* or lesion* or demineralі* or reminerali*))
S19	"Early childhood caries"
S20	"Nursing Bottle Caries"
S21	"Rampant caries"
S22	"Baby bottle tooth decay"
S23	S8 OR S9 OR S10 OR S11 OR S12 OR S13 OR S14 OR S15 OR S16 OR S17 OR S18 OR S19 OR S20 OR S21 OR S22
S24	S7 AND S23
S25	(MH "Child, Preschool")
S26	"Preschool*" or "pre-school*"
S27	"Early childhood*"
S28	toddler*
S29	(MH "Infant")
S30	infant*
S32	S24 AND S31

Note:

1. * denotes truncation
2. # denotes optional wildcard
3. TI focuses on title field
4. AB focuses on abstract field

Scopus

((TITLE-ABS-KEY ("Breast#Feeding" OR "breast#feed*" OR "breast#fed*")) OR (TITLE-ABS-KEY ("Human milk" OR "breast#milk" OR lactat*))) AND ((TITLE-ABS-KEY ((dental* OR tooth* OR teeth* OR oral) W/2 (caries OR cario*))) OR (TITLE-ABS-KEY ("Dental Caries Susceptibility" OR "Tooth demineralі#ation*")) OR (TITLE-ABS-KEY ("Oral health" OR "Dental health")) OR (TITLE-ABS-KEY (dental W/5 (cavit* OR decay* OR lesion* OR demineralі* OR reminerali*))) OR (TITLE-ABS-KEY ((tooth OR teeth) W/5 (cavit* OR decay* OR lesion* OR demineralі* OR reminerali*))) OR (TITLE-ABS-KEY ("Early childhood caries" OR "Nursing Bottle Caries" OR "Rampant caries" OR "Baby bottle tooth decay"))) AND (TITLE-ABS-KEY ("Pre#school*" OR "Early childhood*" OR toddler* OR infant*))

Note:

1. * denotes truncation
2. # denotes wildcard
3. TI focuses on title field
4. AB focuses on abstract field
5. W/n finds two words within a specified limit in any order

Web of science:

1. (TI=(Preschool* or pre-school* or Early childhood* or Toddler* or infant*)) OR AB=(Preschool* or pre-school* or Early childhood* or Toddler* or infant*)
2. (TI=(breast Feed* or breastfeed* or breast-feed* or breast fed* or breastfed* or breast-fed* or Human milk or breastmilk or breast milk or breast-milk or Lactat*)) OR AB=(breast Feed* or breastfeed* or breast-feed* or breast fed* or breastfed* or breast-fed* or Human milk or breastmilk or breast milk or breast-milk or Lactat*)
3. (TI=(((Dental* OR tooth* OR teeth* OR Oral) NEAR/2 (caries OR cario*)) OR Tooth demineralization* OR Oral health* OR Dental health* OR ((dental OR tooth OR teeth) NEAR/5 (cavit* OR decay* OR lesion* OR deminerali* OR reminerali*)) OR (Early childhood caries OR "Nursing Bottle Caries" OR Rampant caries OR "Baby bottle tooth decay").)) OR AB=(((Dental* OR tooth* OR teeth* OR Oral) NEAR/2 (caries OR cario*)) OR Tooth demineralization* OR Oral health* OR Dental health* OR ((dental OR tooth OR teeth) NEAR/5 (cavit* OR decay* OR lesion* OR deminerali* OR reminerali*)) OR (Early childhood caries OR "Nursing Bottle Caries" OR Rampant caries OR "Baby bottle tooth decay").)
4. #1 AND #2 AND #3

Note:

1. * denotes truncation
2. # denotes wildcard
3. TI focuses on title field
4. AB focuses on abstract field
5. Near/n denotes words within ‘n’ words of term

Supplementary Materials S3

Table S2. Critical appraisal of cohort studies.

Studies	Q1	Q2	Q3	Q4	Q5	Q6	Q7	Q8	Q9	Q10	Q11
Abanto et al. 2023	Y	Y	Y	Y	U	Y	Y	Y	Y	Y	Y
Barroso et al. 2021	Y	Y	Y	Y	Y	U	Y	Y	N	Y	Y
Bernabe et al. 2017	Y	Y	Y	Y	Y	U	Y	Y	N	N	Y
Chaffee et al. 2014	Y	Y	Y	Y	Y	Y	Y	Y	Y	Y	Y
Devenish et al. 2020	Y	Y	Y	Y	Y	Y	Y	Y	Y	Y	Y
Feldens et al. 2010	Y	Y	Y	Y	U	Y	Y	Y	Y	Y	Y
Feldens et al. 2018	Y	Y	Y	Y	Y	Y	Y	Y	Y	Y	Y
Haag et al. 2019	Y	Y	Y	Y	Y	Y	Y	Y	Y	Y	Y
Hartwig et al. 2019	Y	Y	Y	Y	Y	Y	Y	Y	Y	Y	Y
Hong et al. 2014	Y	Y	Y	Y	Y	Y	Y	Y	N	N	Y
Nakayama et al. 2022	Y	Y	Y	Y	Y	N	Y	Y	Y	Y	Y
Nirunsittirat et al. 2016	Y	Y	Y	Y	Y	Y	Y	Y	Y	Y	Y
Nunes et al. 2012	Y	Y	Y	Y	Y	U	Y	N/A	Y	Y	Y
Majorana et al. 2014	Y	Y	Y	U	U	Y	Y	Y	Y	U	Y
Manohar et al. 2021	Y	Y	Y	Y	Y	Y	Y	Y	Y	Y	Y
Peltzer et al. 2015	Y	Y	Y	Y	Y	Y	Y	Y	Y	N/A	Y
Peres et al. 2017	Y	Y	Y	Y	Y	Y	Y	Y	N	N	Y
Tanaka et al. 2013	Y	Y	Y	Y	Y	Y	Y	Y	Y	U	Y
Tashiro et al. 2021	Y	Y	Y	Y	Y	N	Y	Y	Y	Y	Y
van Palenstein Helderman et al. 2006	Y	Y	Y	Y	Y	U	Y	N/A	Y	Y	Y
Yokoi et al. 2020	Y	Y	Y	Y	Y	Y	Y	Y	Y	Y	Y
Yonezu et al. 2006	U	Y	U	N	N	N	N	Y	N	U	Y

Note: Y = Yes, N = No, U = Uncertain, N/A = Not applicable.

Checklist questions:

Q1 Were the two groups similar and recruited from the same population?
Q2 Were the exposures measured similarly to assign people to both exposed and unexposed groups?
Q3 Was the exposure measured in a valid and reliable way?
Q4 Were confounding factors identified?
Q5 Were strategies to deal with confounding factors stated?
Q6 Were the groups/participants free of the outcome at the start of the study (or at the moment of exposure)?
Q7 Were the outcomes measured in a valid and reliable way?
Q8 Was the follow up time reported and sufficient to be long enough for outcomes to occur?
Q9 Was follow up complete, and if not, were the reasons to loss to follow up described and explored
Q10 Were strategies to address incomplete follow up utilized?
Q11 Was appropriate statistical analysis used?

Supplementary Materials S4

Table S3. Critical appraisal of case-control studies.

**Studies**	**Q1**	**Q2**	**Q3**	**Q4**	**Q5**	**Q6**	**Q7**	**Q8**	**Q9**	**Q10**
Cvanova et al. 2022	Y	Y	U	Y	Y	Y	Y	Y	Y	Y
Dabawala et al. 2008	Y	Y	Y	U	Y	Y	Y	y	Y	Y
Ganesh et al. 2022	Y	Y	Y	Y	Y	Y	Y	Y	Y	Y
Lima et al. 2016	Y	Y	Y	Y	Y	Y	Y	Y	y	y
Matee et al. 1994	N	U	y	Y	Y	Y	N	Y	Y	Y
Qin et al. 2008	Y	Y	Y	U	Y	Y	Y	Y	Y	Y
Roberts et al. 1994	Y	Y	Y	U	Y	Y	Y	y	y	Y
Seow et al. 2009	N	Y	Y	Y	Y	Y	Y	Y	Y	Y
Werneck et al. 2008	Y	Y	Y	Y	Y	Y	Y	Y	Y	Y

Note: Y = Yes, N = No, U = Uncertain.

Checklist questions

Q1	Were the groups comparable other than the presence of disease in cases or the absence of disease in controls?
Q2	Were cases and controls matched appropriately?
Q3	Were the same criteria used for identification of cases and controls?
Q4	Was exposure measured in a standard, valid and reliable way?
Q5	Was exposure measured in the same way for cases and controls?
Q6	Were confounding factors identified?
Q7	Were strategies to deal with confounding factors stated?
Q8	Were outcomes assessed in a standard, valid and reliable way for cases and controls?
Q9	Was the exposure period of interest long enough to be meaningful?
Q10	Was appropriate statistical analysis used?

Supplementary Materials S5

Table S4. Description of cohort and case-control studies.

Study	Setting/ontext	Participant characteristics	Exposure definition	Outcomes measured	Description of main results	Funding
Abanto 2023 Prospective cohort	Acre, Brazil<br><br>Setting: Clinical	Recruitment age: birth<br>Follow-up: 6, 12, 24 months<br><br>1246 included in the study, 800 Analysed<br><br>Gender of participants not reported	Breastfeeding: <12 months, 12-23 months, ≥24 months	Caries description: ECC-dmft index (WHO criteria)<br>Cavitated lesions extending to dentin<br><br>Clinical examination conducted by 2 trained and calibrated paediatric dentists. Intra- and inter-examiner kappa coefficient = >0.94.	Weak association between prolonged BF and dental caries.<br>BF for 12–23<br>Months: PR=2.13, 95% CI (1.46–3.11), p<0 .001<br>BF for ≥24 months: PR=3.21, 95% CI (2.12–4.87), p< 0.001	Brazilian National Council for Scientific and Technological Development (CNPq) and São Paulo Research Foundation
Barosso 2021 Prospective cohort (nested in cross-sectional)	Diamantina, Brazil<br><br>Setting: Clinical	Recruitment age: 2-3 yrs<br>Follow-up: after 3 yrs<br><br>138 included in the study,<br>132 analysed<br><br>59 males,<br>73 females	Breastfeeding: ≤24 months, >24 months<br>Bottle feeding: ≤24 months, >24 months	Caries description: S-ECC<br>ICDAS scores:<br>1-2: Initial caries<br>3-4: Established caries<br>5-6: Severe caries<br><br>Clinical examination conducted by two trained and calibrated examiners. Intra-examiner Kappa coefficient = 0.85 (minimum) and inter-examiner Kappa co-efficient = 0.86 (minimum)	BF >24 months is a risk factor for severe dental caries in preschool children<br><br>BF > 24 months: RR adjusted for child's habits =2.62, 95% CI (1.42- 4.84)<br>BF> 24 months: RR adjusted for clinical factors = 2.24, 95% CI (1.23- 4.08)	Not reported

Bernabe. 2017 Prospective cohort	Dundee, Scotland<br><br>Setting: Field	Recruitment age: 1 yr<br>Follow-up: 2, 3 and 4 yrs<br><br>1419 included in the study,<br>1102 analysed<br><br>592 males,<br>510 females	Breastfeeding: Never, <6months ≥6 months	Caries description: ECC mean dmfs, cavitated & non-cavitated lesions<br><br>Caries diagnosed by a trained examiner. Intra-examiner Cohen's Kappa co-efficient = 0.75	No association between BF & ECC.<br><br>LME model with main effects:<br>BF < 6 months: Coef = 0.02, 95% CI (-0.23-0.28)<br>BF ≥ 6 months: Coef = 0.06, 95% (-0.25-0.37)	Chief Scientist Office of the Scottish Office Department of Health
Chaffee. 2014 Prospective cohort (nested)	Porto Alegre, Brazil<br><br>Setting: Field	Recruitment age: birth<br>Follow-up: 6, 12 and 38 months<br><br>715 included in study,<br>458 analysed<br><br>333 males,<br>332 females	Breastfeeding: <6 months, 6-11 months, 12-23 months, >24 months	Caries description: dmfs (WHO criteria),<br>S-ECC=≥1 maxillary anterior teeth surface or ≥4 dmfs<br><br>Clinical examination conducted by two dentists. Intra-examiner Kappa coefficient<br>= 0.83 and inter-examiner Kappa co-efficient = 0.75	Highest S-ECC in children BF for ≥ 24 months.<br>BF for 6-11 months: aPR = 1.77, 95% CI (1.12 - 2.85)<br>BF for 12- 23 months: aPR = 1.82, 95% CI (0.85 - 3.20)<br>BF for >24 months: aPR = 2.10, 95% CI (1.5 - 3.25)<br>Prolonged and frequent BF increases caries risk. Frequent day time BF for >24 months (aPR) = 1.38, 95% CI (0.38 - 0.58)	Not reported
Cvanova 2022 Case-control	South Moravia, Czech Republic<br><br>Setting: Clinical	Recruitment age: <6yrs<br>Follow-up: N/A<br><br>Recruited:<br>n = 632<br><br>Met the inclusion criteria:<br>n = 414	Breastfeeding: ≤ 6months, > 6 months<br>BF at night: yes/no	Caries assessment: S-ECC dmft<br><br>Cases (dmft≥ 6) i.e. S-ECC according to AAPD<br>Controls (dmft=0, > 2yrs old)<br>Children with dmft ≥ 1 or ≤ 5 were excluded<br><br>Clinical examination	BF ≤ 6 months increases the risk for sECC (aOR = 2.71; 95%CI- 1.45-5.07; p = 0.002)	Research: Ministry of Health of the Czech Republic and University Hospital Brno.<br>Publication: European Union

		Cases n = 214<br>Controls n = 200<br><br>Data analysed<br>n = 311<br>Cases = 164<br>Controls = 147<br><br>169 males,<br>142 females		conducted by dentists<br>Number of examiners and calibration not reported<br>No kappa agreement reported		
Dabawala 2017<br>Case-control	Mangalore, India<br><br>Setting: Field	Recruitment age: 3-5 yrs<br>Follow-up: N/A<br><br>Contacted: n = 605<br>Consented n = 550<br><br>Data analysed n = 422<br>Cases n = 211<br>Controls n = 211<br><br>197 males,<br>225 females	Feeding method: Breastfeeding, Bottle feeding, Mixed<br><br>Feeding duration:<br>< 1 year, > 1 year	Caries description: dmfs<br><br>Cases were defined as per AAPD definition of ECC<br>Cases (dmfs ≥ 1)<br>Controls (dmfs = 0)<br><br>Clinical examination conducted by two trained and calibrated dentists intraclass correlation coefficient<br>= 0.95	BF or bottle feeding >12 months increased the risk for ECC (aOR = 3.93; 95%CI- 1.68-9.17; p = 0.002)	None
Devenish. 2020<br>Cohort	Adelaide, Australia	Recruitment age: birth<br>Follow-up age: between 2-3 yrs<br><br>1039 included in the study,<br>965 analysed	Breastfeeding:<br><1 month, 1 to <6 months, 6 to <12 months, ≥ 12 months<br>Nocturnal feeding:<br>Mixed feeding, Bottle feeding	Caries description:<br>mean dmfs, cavitated & non-cavitated lesions<br>ECC = dmfs≥ 1<br><br>Clinical examination was carried out by a team of trained and calibrated dentists. Cohen's Kappa	No association between BF > 12 months & ECC relative to BF for 6-12 months. PR = 1.42; 95% CI (0.85- 2.38)<br><br>No association between feeding to sleep:<br>BF: PR = 1.12; 95% CI (06.7- 1.88)<br>Bottle-feeding: PR = 0.66; 95% CI (0.37–1.16)	Australian Government Research Training Program Scholarship

		Gender of participants not reported	only, Breastfeeding only, none	coefficient not reported.		
Feldens. 2010 Prospective cohort (nested in RCT)	Sao Leopoldo,Brazil<br><br>Setting: Field	Recruitment age: birth<br>Follow-up age: 1 yr, 4 yrs<br><br>500 included in the study,<br>340 analysed<br><br>195 males,<br>145 females	Breastfeeding duration:<br><12 months, ≥12 months	Caries description:<br>S-ECC: ≤1 maxillary anterior teeth surface or dmfs 5≥<br><br>Clinical examination conducted by the same examiner on the 1st and 4th year assessments. Intra-examiner kappa coefficient = 0.90	BF ≥12 months crude RR = 1.62; 95% CI (1.21- 2.17)	Not reported
Feldens. 2018 Prospective cohort (nested in RCT)	Porto Alegre, Brazil<br><br>Setting: Not reported	Recruitment age: birth<br>Follow-up: 6, 12 and 38 months<br><br>458 included in the study,<br>345 analysed<br><br>172 males,<br>173 females	Breastfeeding and bottle-feeding frequency	Caries description: d1mf and d1mfs,<br>ECC ≥1<br>S-ECC: ≥1 maxillary incisors affected or d1mfs ≥4 or for children <36 months ≥1 smooth surface caries<br><br>Clinical examination conducted by two trained and calibrated dentists. Intra-examiner Kappa coefficient = 0.83 and inter-examiner Kappa co-efficient = 0.75	Positive association between high frequency feeding (breast and/or bottle) in late infancy and ECC.<br>High frequency BF: aRR = 1.82; 95% CI (1.28- 2.57)	The NIH National Institute for Dental and Craniofacial Research, the NIH National Centre for Advanced Translational Sciences, the Rio Grande do Sul Research Support Foundation and the Coordination for the Improvement of Higher Education Personnel

Ganesh<br>2022<br>Case-control	Chennai, India<br><br>Setting: Field	Recruitment age: 12-36 months<br>Follow-up: N/A<br><br>Contacted: n = 627<br>Consented n = 627<br><br>Data analysed n = 627<br>Cases n = 302<br>Controls n = 325<br><br>Gender of participants not reported	Sleep-time feeding practices:<br>Beginning of sleep,<br>early morning hours sleep,<br>course of sleep<br>Feeding mode: breast, bottle or other feeding modes (sipper, tumbler, cup, etc.)	Caries description: ICDAS criteria<br><br>Clinical examination conducted by trained dentist who was supervised by an experienced researcher.<br>No intra-examiner kappa agreement reported	Strong association between sleep-time BF/ bottle feeding and ECC. BF at the beginning of sleep (aOR = 6.70; 95%CI- 4.20- 10.70; p = 0.001) and during the course of sleep (aOR = 6.50; 95%CI- 2.80- 15.00; p = 0.001) increased the risk for ECC. Bottle feeding at the beginning of sleep (aOR = 5.10; 95%CI- 3.10- 8.30; p = 0.001) and during the course of sleep (aOR = 8.32; 95%CI- 5- 13.84; p = 0.001) increased the risk for ECC. Duration of feeding and sweet additives to milk increased the risk of ECC.	Sri Ramchandra Institute of Higher Education and Research
Haag<br>2019<br>Prospective cohort (nested in RCT)	South Australia<br><br>Setting: Field	Recruitment age: birth<br>Follow-up age: 2 yrs, 3 yrs,<br><br>448 included in the study,<br>307 analysed<br><br>160 males,<br>147 females	Breastfeeding:<br>Never, <12 months,<br>12-23 months,<br>≥24 months	Caries description: ECC - dmfs and dfs, cavitated and non-cavitated lesions<br>D1: demineralization only<br>D2: lesions in the enamel only<br>D3: lesions in the enamel and dentine	Compared to children who were never breastfed, prevalence of ECC was lower than those who were breastfed <12 months (33.1% vs 25.7%). Prevalence of ECC in children breastfed for 12-23 months was 37%. Children breastfed for ≥24 months had the highest prevalence (56.4%) with a mean dmfs of 6.6. aOR = 5.22; 95% CI (2.06- 8.39)	National Health and Medical Research Council of Australia
Hartwig<br>2019<br>Retrospective cohort	Pelopotas, Brazil<br><br>Setting: Clinical	Recruitment age: <1yrs<br>Follow-up age: 3 yrs<br><br>325 included in the study,	Breastfeeding:<br><6 months, 6-11 months, 12-23 months, >24 months	Caries description: ECC - mean dmfs, cavitated & non-cavitated lesions<br>ECC ≥1<br><br>Clinical examination conducted by trained	More cases of dental caries in children breastfed for ≥24months. aRR = 8.29; 95% CI (1.82- 37.72)	Not reported

		325 analysed<br><br>165 males,<br>160 females		paediatric dentists and dental students. Calibration and kappa coefficient not reported		
Hong<br>2014<br>Prospective cohort	Iowa, USA<br><br>Setting: Clinical	Recruitment age: birth<br>Follow-up: 5 yrs, 9 yrs<br><br>698 included in the study,<br>509 analysed<br><br>249 males,<br>260 females	Breastfeeding: <6 months, 6-12 months, >12 months	Caries description: ECC - D1-D3: D1- demineralisation only, D2- cavitated enamel, D3- cavitated dentin.<br>Caries measured as dfs (decayed or filled surface) on primary second molars<br><br>Clinical examination conducted by three trained and calibrated dentists. Inter-examiner kappa coefficient for dfs at 5 years old = 0.90	Prolonged BF decreases the risk of ECC in preschool children.<br>OR for primary 2nd molar caries experience at 5 years old=15.58; 95% CI; p = 0.005	NIH
Lima<br>2016<br>Case-control	Piaui, Brazil<br><br>Setting: Clinical	Recruitment age: 1yr - ≤5 yrs<br>Follow-up: N/A<br><br>Recruited: n = 3,374<br><br>Data analysed n = 530<br>Cases n = 267<br>Controls n = 263<br><br>267 males,<br>263 females	Breastfeeding: < 6 months, ≥ 6 months<br><br>Nocturnal breastfeeding: ≤ 16 months, > 16 months	Caries description: dmfs<br>Cavitated and non-cavitated lesions<br>ECC as per AAPD<br>Cases (dmfs ≥ 1)<br>Controls (dmfs = 0)<br><br>Clinical examination conducted by one examiner. Intra-examiner error measured by kappa coefficient = 0.90	BF ≤ 16 months decreases the risk for ECC (OR = 0.51; 95%CI- 0.39- 0.65; p <0.001)	Brazilian National Council for Scientific and Technological Development (CNPq)

Majorana 2014<br>Retrospective cohort	Besica, Italy<br><br>Setting: Clinical	Recruitment age: birth<br>Follow-up: 24-30 months<br><br>2450 included in the study,<br>2395 analysed<br><br>1181 males,<br>1214 females	Feeding practice:<br>Exclusive BF,<br>Moderate to high mixed feeding,<br>Low mixed feeding,<br>Exclusive formula feeding	Caries description: ICDAS II, Cavitated and non-cavitated lesions<br>ICDAS scores:<br>1: visual change in enamel after air drying<br>2: frank visual change in the enamel<br>3: Cavitation in enamel only<br>4: shadowing in the dentine<br>5: Cavitation in the dentine<br>6: Extensive cavity into the dentine<br><br>Clinical examination conducted by two calibrated dentists.<br>Inter-examiner Kappa coefficient = 0.84	Formula fed infants showed more severe caries than exclusively breastfed or moderate-high mix fed infants.<br>OR for various feeding practices with ICDAS scores = 6.75,<br>95% CI (6.00-7.58)	None
Manohar 2021<br>Cohort	Australia<br><br>Setting: Clinical	Recruitment age: 4-6 weeks<br>Follow-up: 4 months, 8 months, 1 year, 2 years, 3 years<br><br>934 included in the study, 718 analysed<br><br>372 males,<br>346 females	Breastfeeding duration:<br>< 4 months;<br>4 - < 6months,<br>6 - <12 months;<br>≥ 12 months	Caries description: ECC dmfs<br><br>Clinical examination conducted by trained and experienced dental therapists in clinical setting. No intra- and inter-examiner kappa agreement reported	Prolonged breastfeeding (≥ 12 months) was associated with ECC (aIRR = 2.17, 95% CI: 1.27–3.73)	NHMRC, NSW Health, Australian Dental Research Foundation, Western Sydney University, and Oral Health Foundation

Matee<br>1994<br>Case-control (nested)	Tanzania<br><br>Setting: Field	Recruitment age: 1-4 yrs<br>Follow-up: N/A<br><br>Met the inclusion criteria:<br>n = 459<br>Cases n = 153<br>Controls n = 306<br><br>Data analysed n = 359<br>Cases n = 116<br>Controls n = 243<br><br>Gender of participants not reported	Breastfeeding duration<br><br>Nocturnal feeding:<br>Never, occasionally, always<br><br>Duration of nipple in the mouth: 0 h, ½ h, 1 h, >1 h<br><br>Bottle feeding and content in bottle	Caries description: dmfs<br>Cavitated lesions only<br>ECC: caries on ≥2 maxillary incisors<br><br>Clinical examination conducted by one examiner following WHO guidelines. No intra-examiner kappa agreement reported	Risk factor for ECC:<br>Duration of BF (1yr vs. 3 yrs) OR = 2.4 (95% CI 0.7-9.1), p = 0.18. Night-time breastfeeding (0 vs. 5) OR = 17.8 (95% CI 6.3-50.3) p<0.0001	Not reported
Nakayama<br>2022<br>Prospective cohort	Hokkaido, Japan<br><br>Setting: Clinical	Recruitment age: 18-23 months<br>Follow-up: 3 yrs<br>1006 included in the study, 872 analysed<br><br>486 males,<br>520 females	Breastfeeding:<br><18 months, ≥ 18 months<br>Nocturnal BF vs no nocturnal BF	Caries description: ECC dmft index (WHO criteria)<br><br>Clinical examination carried out by 40 dentists with over 10 years of experience. Examiner calibration was not done.	Risk factors for developing ECC at three years of age were nocturnal BF, prolonged BF, dental caries at 18-23 months of age, frequency of snacking, less parent supervised toothbrushing, sharing of utensils among parents and child.<br><br>High risk of ECC with nocturnal breastfeeding, OR = 3.59; 95% CI (2.41-5.36)	Not reported
Nirunsittirat<br>2016<br>Prospective cohort	Thailand<br><br>Setting: Field	Recruitment age: 28-38 wks IUL<br>Follow-up: 3-4 yrs	Breastfeeding duration:<br><6 months, 6-11 months, 12-17	Caries description: ECC - dmfs index (WHO criteria)<br><br>Clinical examination	BF duration (number of children):<br>< 6 months: 121<br>6- 11 months: 87<br>12- 17 months: 179	Khon Kaen University Research grants

		860 included in the study,<br>544 analysed<br><br>273 males,<br>271 females	months, ≥18 months	conducted by two calibrated examiners. Inter- and intra-examiners agreement = >90%	≥ 18 months: 157<br><br>Nocturnal feeding (number of children):<br>Never: 155<br>1-3 times: 66<br>More than 3 times: 121<br><br>BF duration and nocturnal feeding not included in the multivariate analysis	
Nunes<br>2012<br>Retrospective cohort	Brazil<br><br>Setting: Field	Recruitment age: 18-42 months<br>Follow-up: N/A<br><br>260 included in the study,<br>241 analysed<br><br>Gender of participants not reported	Nocturnal breastfeeding: Present/ Absent	Caries description: ECC-dmft (WHO criteria), cavitated lesions only<br><br>Clinical examination carried out by one calibrated examiner. intra-examiner kappa coefficient = 0.91	Prolonged breast-feeding was not associated with ECC, using a hierarchical approach (IDR 1.15; 95%CI 0.84–1.59; P = 0.363).	FAPEMA (Maranha˜o Research and Scientific and Technological Development Foundation), DECIT/ SCTIE/MS (Department of Science and Technology, Ministry of Health), and CNPq (National Council for Scientific and Technological Development)
Peltzer<br>2015<br>Prospective cohort	Mueang Nan, Thailand<br><br>Setting: Field	Recruitment age: 28- 38 wks IUL<br>Follow-up age: 3 yrs<br><br>783 included in the study,<br>597 analysed	Breastfeeding duration:<br>Never,<br>< 4months; ≥ 4 months<br><br>Nocturnal BF at 12 months:	Caries description: dmfs (WHO criteria),<br>S-ECC: dmfs ≤ 1 maxillary anterior teeth surface or dmfs ≥ 4<br><br>Clinical examination carried out by three trained and	Risk factors including environmental factors and risky behaviour such as sleeping with bottle at 30 months identified to cause S-ECC.<br>Compared to never breastfed, BF was protective against ECC. aOR = 0.63; 95% CI (0.38–1.04); p < 0.01<br>Compared to children who didn't BF	Not reported

		299 males,<br>298 females	Present/ Absent	calibrated examiners. Kappa coefficient not reported.	at night, children who BF at night: uORr = 1.61; 95% CI (0.77- 3.38); p ≤ 0.25	
Peres<br>2017<br>Prospective cohort (nested)	Pelotas, Brazil<br><br>Setting: Field	Recruitment age: Birth<br>Follow-up: 3 months, 12 months, 4 yrs<br><br>1303 included in the study,<br>1129 analysed<br><br>Gender of participants not reported	Breastfeeding: ≤ 12 months, 13-23 months, ≥ 24 months	Caries description: average dmfs (WHO criteria)<br>S-ECC: ≥6<br><br>Clinical examination carried out by eight trained and calibrated examiners. Kappa coefficient = 0.92	Positive association between S-ECC and BF ≥ 24 months. RR in children BF ≥ 24 months is 2.4 times more than children BF < 12monthS or BF 12-23 months. (Duration of BF ≥24 months vs. ≤12 months, RR=2.4, 95% CI; 1.7-3.3)	Brazilian National Council for Scientific and Technological Development (CNPq)
Qin<br>2008<br>Case-control	Beijing, China<br><br>Setting: Clinical	Recruitment age: <4 yrs<br>Follow-up: N/A<br><br>Recruited n=514<br><br>Data analysed n = 246<br>Cases n = 117<br>Controls n = 129<br><br>Gender of participants not reported	Feeding habit: Breastfeeding, Milk without sugar, milk with sugar<br><br>Feeding duration: < 6 months, 6-12 months<br><br>Sleeping while feeding after 12 months old: Present/ Absent	Caries description: dmft (WHO criteria), S-ECC > 5<br><br>Cases (dmft > 5) i.e. S-ECC according to AAPD<br>Controls (dmft=0)<br>Children with dmft ≥ 1 or ≤ 5 and enamel hypoplasia were excluded<br><br>Clinical examination conducted by two calibrated paediatric dentists. Kappa agreement reported	Nocturnal feeding is one of the risk factors for S-ECC (66% of S-ECC children drank milk at night, p<0.001)	Beijing Medical Research and Development

Roberts<br>1994<br>Case-control	South Africa<br><br>Setting: Not reported	Recruitment age: 1-4 yrs<br>Follow-up: N/A<br><br>Recruited n = 1,263<br>Data analysed n = 218<br>Cases n = 109<br>Control n = 109<br><br>Gender of participants not reported	Feeding history: Breast, Bottle<br><br>Frequency of feeding:<br>Day, night<br><br>Duration of BF: any, demand, scheduled	Caries description: ECC ≥ 2 decayed labial or palatal surfaces of maxillary deciduous incisors, based on definition of Beal and James (1970) and of Winter et al. (1971).<br>Cases (ECC ≥ 2)<br>Controls (ECC <2)	No association between ECC and length or type of feeding.<br>(χ^2 =0.801, df = 1, P< 0.50 > 0.25)	Not reported
Seow<br>2009<br>Case-control	Queensland,<br>Australia<br><br>Setting: Field	Recruitment age: 0-4 yrs<br>Follow-up: N/A<br><br>Data analysed: n=617<br>Cases: n = 156<br>Controls: n = 461<br><br>Gender of participants not reported	Feeding history: Breast, Bottle or both	Caries description: dmft (WHO criteria), ECC >1 dmft<br>Cases (dmft≥ 1)<br>Controls (dmft=0)<br><br>The inter- and intra-examiner were consistent with 92-95% reproducibility.	No risk associated with BF and bottle-feeding until 2 years of age. Childcare ECC vs. Controls OR = 0.41; 95%CI- 0.13- 1.29; p = 0.048(< 0.05); public clinic ECC vs. controls<br>OR = 0.25; 95%CI- 0.08- 0.84; p = 0.048(<0.05)	Health Promotion Queensland
Tanaka<br>2013<br>Prospective cohort	Osaka,<br>Japan<br><br>Setting: Not reported	Recruitment age: birth<br>Follow-up: 41- 50 months old<br><br>Consent received:	Breastfeeding duration: < 6 months, 6-11 months, 12-17 months, ≥1 8 months	Caries description: dmft (WHO criteria), ECC: ≥ 1 dft<br>Moderate ECC = 1 – 4 dft an no caries in maxillary incisors<br>Severe ECC = dft ≥ 1and	Prolonged BF one of the risks factors associated with ECC. aOR for BF ≥ 18 months = 2.47; 95% CI (0.95- 6.59)<br><br>aOR for moderate ECC and S-ECC for:<br>BF 6-11 months = 0.72, 95% CI (0.25-	Kakenhi, HEalth and Labour Sciences research grants, Research onAllergic Disease and Immunology from

		1002<br>No. of participants in all the surveys: 494<br>318 included in the study, 315 analysed<br><br>Gender of participants not reported		caries in the maxillary anterior teeth or dft ≥ 5<br><br>Clinical examination conduced by dental hygienists. Training and calibration of the examiners not reported. Kappa coefficient not reported.	2.02) and 0.41; 95% CI (0.16- 4.01) respectively<br>BF 12-17 months = 1.23, 95% CI (0.44- 3.52) and 0.81; 95% CI (0.16- 4.01) respectively<br>BF≥ 18 months = 2.70; 95% CI (0.88- 8.66) and 2.30; 95% CI (0.47- 12.03) respectively	the Ministry of Health, Labour and Welfare, Tokyo, Japan
Tashiro 2021<br>Prospective cohort	Tokyo, Japan<br><br>Setting: Clinical	Recruitment age: 18-19 months<br>Follow-up age: 3 yrs<br>414 included in the study, 387 analysed<br><br>200 males,<br>187 females	BF at 18 months: Present/ Absent<br><br>BF and/or bottle feeding in bed: Present/ Absent	Caries description: Presence or absence of caries. Cavitated and non-cavitated lesions<br><br>Clinical examination conducted by a trained paediatric examiner. Kappa coefficient not reported.	Non-significant association between nocturnal BF and/ or bottle feeding.<br>BF at 18 months (No vs. Yes): aOR = 7.10; 95% CI (2.85- 19.45); p < 0.001<br>Nocturnal feeding (breast and/or bottle) aOR = 2.072; 95% CI (0.986- 4.368)<br><br>-	Not reported
van Palenstein Helderman 2006<br>Retrospective cohort	Daik-U, Myanmar<br><br>Setting: Field	Recruitment age: 25- 30 months<br>198 included in the study,<br>163 analysed<br><br>84 males,<br>79 females	Total exposure time to BF (low/high)<br>Duration of BF<br>Breast nipple in child's mouth at night	Caries description: Frank cavitation<br>ECC: ≥1 carious teeth<br><br>Clinical examination conducted by one dentist. Intra-examiner kappa coefficient = 1.	Compared to infants who did not breast feed at night for >2 times, infants who did had the OR = 35; 95%CI (6-186)	Dental Health International Netherlands
Werneck 2008<br>Case-control	Toronto, Canada<br><br>Setting: Not	Recruitment age: 48 months<br>Follow-up age: N/A	Feeding history: Breastfed or bottle-fed	Caries description: dmft (WHO criteria).<br>Cases were defined as per AAPD definition of ECC	- BF increases the risk of ECC (OR = 5.32; 95%CI- 1.64- 17.24; p = 0.003). No significant association with the duration of BF.	Not reported

	reported	Recruited: n = 148<br><br>Met inclusion criteria and analysed: n = 104<br>Cases: n = 52<br>Controls: n = 52<br><br>106 males,<br>94 females		Cases (dmfs ≥ 1)<br>Controls (dmfs = 0)<br><br>Clinical examination conducted by single examiner calibrated by paediatric dentist. No intra-examiner kappa agreement reported		
Yokoi<br>2020<br>Prospective cohort	Maniwa city, Japan<br><br>Setting: Field	Recruitment age: 18 months<br>Follow-up age: 3 yrs<br><br>806 included in the study, 640 Analysed<br><br>Gender of participants not reported	Prolonged BF: Present/ Absent	Caries description: dmft (WHO criteria); new ECC (ECC>0)<br><br>Clinical examination conducted by 38 trained dentists. Kappa coefficient not reported.	Prolonged BF significantly increases risk of ECC OR=1.71; 95%CI (1.15–2.55); $p < 0.001$	Not reported
Yonezu<br>2006<br>Prospective cohort	Tokyo, Japan<br><br>Setting: Not reported	Recruitment age: 18 months<br>Follow-up: 24 months, 36 months<br>1120 children examined<br>592 included in the study,<br>592 analysed	Breast vs. Bottle feeding at 18 months, 24 months and 36 months	Caries description: Presence or absence of caries. Cavitated and non-cavitated lesions	Dental caries more prevalent in children were breastfed at 18 moths than their counter parts who were weaned off before 18 months. Prolonged bottle fed children were less likely to have dental caries than prolonged breastfed children.<br><br>Mean dft at 18 months:<br>Breastfed: 0.36	Not reported

		(additional 205 controls)<br><br>Gender of participants not reported			Bottle-fed: 0.17<br>Control: 0.06<br><br>Mean dft at 24 months:<br>Breastfed: 0.51<br>Bottle-fed: 0.34<br>Control: 0.11<br><br>Mean dft at 36 months:<br>Breastfed: 1.27<br>Bottle-fed:0.85<br>Control: 0.54	

BF: Breastfeeding; ECC: Early childhood caries, S-ECC: Severe early childhood caries, PR = Prevalent Risk, RR = relative risk, OR = Odds Ratio, LME = Linear Mixed Effects, IRR = Incidence rare ratio, ICDAS = International Caries Detection and Assessment System, AAPD- = American Academy of Paediatric Dentistry, WHO = World Health Organisation, N/A: Not applicable

Supplementary Materials S6. Excluded Studies

1. Alaluusua 1990 Cross sectional study
2. al-Dashti 1995 Cross sectional study
3. Azevedo 2005 Cross sectional study
4. Bahuguna 2013 Outcome measured up to 18 years of ageStudy Reason for exclusion
5. Bankel 2011 There is no clarity in the study methodology. Restored and extracted teeth due to caries were reported and def or di were calculated.
6. Beckett 2022 Inclusion criteria not met. Mean age of children = 6.6 years
7. Birungi 2015 The study is testing the effect of individual home-based peer counselling on breastfeeding and caries
8. Blanco 2021. Not in English
9. Boustedt 2018 Reason for exclusion: outcome not related to exposure.
10. Caetano 2010 This study does not have relevant comparative groups as it is assessing the feeding practices and dietary intake of healthy infants.
11. Campus 2009 National cross-sectional study
12. Carino 2003 Cross sectional study
13. Carvajal Roca 2020 Not in English
14. Chattopadhyay 2020 Cross sectional study
15. Chiao 2021 Cross sectional study
16. Conway 2023. Comment on a paper
17. Dini EL 2000 Cross sectional study
18. Du 2000 Cross sectional study
19. Du 2007 Cross sectional study
20. Dye 2004 National cross-sectional study
21. Eronat 1992 Data only provided for breastfeeding duration for study group and not control group.
22. Faye 2006 Cross sectional despite reported as cohort study by the author
23. Feldens 2007 This study does not have relevant comparative groups as it is assessing the effectiveness of home visits for advising mothers about breast feeding and weaning on early childhood caries (ECC) at the age of 12 months
24. Feldens 2010b This study does not have relevant comparative groups as it is testing the effectiveness of home visits advising mothers about healthy feeding practices during the first year of life on the occurrence of early childhood caries and severe early childhood caries at 4 years of age.
25. Folayan 2010 Cross sectional study
26. Forsman 1974 Cross sectional study
27. Hallett 2003 Cross sectional study
28. Hallonsten 1995 Cross sectional study
29. Haq 1985 Cross sectional study
30. Hardy 1978 Cross sectional study
31. Harrison 1997 Cross sectional study
32. Holt 1982 Cross sectional study
33. Hu 2019 Exposure criteria not met
34. Huntington 2002 No clear distinction between breastfeeding and no breastfeeding. Appears that the breastfeeding group also received bottle feeding.
35. Iida 2007 Cross sectional study
36. Johansson 2010 Cross sectional study
37. Kato 2015 Self-reported outcome measures by mother and number of dental caries not reported.
38. Kramer 2007 Reports impact of exclusive breastfeeding on permanent teeth so not included.
39. Kramer 2009 Reports the impact of exclusive breastfeeding on permanent teeth so not included.
40. Kubota 2020 Cross sectional study
41. Livny 2007 Cross sectional study
42. Masumo 2012 Cross sectional study

43. Mattos-Graner 1998 Cross sectional study
44. Nishimura 2008 Caries activity test score is an outcome measure which is not relevant to this review.
45. Nobile 2014 Cross sectional study
46. Novak 1965 This study does not have relevant comparative groups. Only assesses dental caries In children who were beastfed and artificially fed during the first month of their life.
47. Oliveira 2006 Retrospective cohort study. Focus on influence on enamel defects in the development of dental caries.
48. Ollila 2007 Outcomes measured at 7 years.
49. Olatossi 2021 Cross sectional study
50. Othman 2021 Cross sectional study
51. Onur 2021 Cross sectional study
52. Oulis 1999 Nursing caries (case) defined as having at least two anterior maxillary teeth affected with caries. Non-nursing caries (control) defined as having no more than one maxillary anterior carious tooth.
53. Park 2022 Cross sectional study
54. Peltzer 2014 Dental caries expressed as percentage of increment. *Outcome criteria*
55. Perera 2014 Cross sectional study
56. Prakash 2012 Cross sectional study
57. Priesnitz 2016 Cross sectional study
58. Qadri 2012 Cross sectional study
59. Qin 2022 Not in English
60. Retnakumari 2012 Cross sectional study. Study Reason for exclusion
61. Roberts 1993 Cross sectional study
62. Rosenblatt 2004 Cross sectional study
63. Sankeshwari 2012 Cross sectional study
64. Santos 2002 Cross sectional study
65. Sayegh 2002 Cross sectional study
66. Sayegh 2005 Cross sectional study
67. Schluter 2007 Filling and extraction experiences are reported as outcome measure which is not relevant to this review.
68. Serwint 1993 Cross sectional study
69. Severino 2021 Cross sectional study
70. Shrutha 2013 Cross sectional study
71. Silver 1987 The study has a follow period up to 8–10 years.? Need to check:3 years and 8-10 years
72. Slabsinskiene 2010 Cross sectional study
73. Songo 2013 Cross sectional study
74. Tada 1999 Results reported as caries increment.
75. Tanaka 2012 Cross sectional study
76. Thitasomakul 2006 Dental caries not associated with breastfeeding
77. Thitasomakul 2009 Retrospective cohort study that reports Crude caries increment and incidence density ratio and not rate of dental caries.
78. Tiano 2009 Cross sectional study
79. Tyagi 2008 Cross sectional study
80. Vachirarojpisan 2004 Cross sectional study
81. van Meijeren-van Lunteren 2021. Inclusion criteria not met. Dental imaging used instead of dental examination for assessment of caries, mean age of participants at dental imaging = 6.1 years
82. Vazquez-Nava 2008 Cross sectional study
83. Vitolo 2005 This study does not have relevant comparative groups as it is assessing the impact of an intervention "Ten Steps to Healthy Feeding" which is a nutritional guide for children under 2 on nutritional conditions and infant health in low-income families.
84. Wendt 1995 Cross sectional study

85. Wong 2017 Cross sectional study
86. Yewei 2001 Cross sectional study where children were examined in a field survey and grouped into cases and controls
87. Yonezu 1998 exposure criteria not met.
88. Yonezu 2006b Cross-sectional study
